# Systematic pulmonary embolism follow-up increases diagnostic rates of chronic thromboembolic pulmonary hypertension and identifies less severe disease: results from the ASPIRE Registry

**DOI:** 10.1183/13993003.00846-2023

**Published:** 2024-02-01

**Authors:** Charlotte Durrington, Judith A Hurdman, Charlie A Elliot, Rhona Maclean, Joost Van Veen, Giorgia Sacccullo, Duneesha De-Foneska, Andrew J Swift, Smitha Rajaram, Catherine Hill, Steven Thomas, Krit Dwivedi, Samer Alabed, James M Wild, Athanasios Charalampopoulos, Abdul Hameed, Alexander MK Rothman, Lisa Watson, Neil Hamilton, A A Roger Thompson, Robin Condliffe, David G Kiely

**Affiliations:** 1 Sheffield Pulmonary Vascular Disease Unit, Royal Hallamshire Hospital, Sheffield Teaching Hospitals NHS Foundation Trust, Sheffield, United Kingdom; 2 Division of Clinical Medicine, University of Sheffield, Sheffield, United Kingdom; 3 Department of Haematology, Royal Hallamshire Hospital, Sheffield Teaching Hospitals NHS Foundation Trust, Sheffield, United Kingdom; 4 Department of Radiology, Royal Hallamshire Hospital, Sheffield Teaching Hospitals NHS Foundation Trust, Sheffield, United Kingdom; 5 Insigneo Institute, University of Sheffield, Sheffield, United Kingdom; 6 National Institute for Health and Care Research Sheffield Biomedical Research Centre, Sheffield, UK

## Abstract

**Background:**

Diagnostic rates and risk factors for the subsequent development of chronic thromboembolic pulmonary hypertension (CTEPH) following pulmonary embolism (PE) are not well defined.

**Methods:**

Over a 10-year period (2010-2020), consecutive patients attending a PE follow-up clinic in Sheffield (population 554,600) and all patients diagnosed with CTEPH, at a PH referral centre in Sheffield (referral population estimated 15-20 million) were included.

**Results:**

Of 1956 patients attending the Sheffield PE clinic 3 months following a diagnosis of acute PE, 41 were diagnosed with CTEPH with a cumulative incidence of 2.10%, with 1.89% diagnosed within 2 years. Of 809 patients presenting with pulmonary hypertension and diagnosed with CTEPH, 32 were Sheffield residents and 777 non-Sheffield residents. Patients diagnosed with CTEPH at the PE follow-up clinic had shorter symptom duration (p<0.01), better exercise capacity (p<0.05) and less severe pulmonary haemodynamics (p<0.01), compared to patients referred with suspected pulmonary hypertension. Patients with no major transient risk factors present at the time of acute PE had a significantly higher risk of CTEPH compared to patients with major transient risk factors, OR of 3.6 (95% CI 1.11 to 11.91, p=0.03), The presence of 3 CT features of pulmonary hypertension in combination with ≥2 of 4 features of chronic thromboembolic pulmonary disease (CTEPD) at the index PE was present in 19% of patients who developed CTEPH and in 0% of patients who did not. Diagnostic rates and pulmonary endarterectomy rates were higher at 13.2/million/year and 3.6/million/year for Sheffield residents compared to 3.9–5.2/million/year and 1.7–2.3/million/year for non-Sheffield residents, respectively.

**Conclusions:**

In the real world setting a dedicated PE follow-up pathway identifies patients with less severe CTEPH and increases population-based CTEPH diagnostic and pulmonary endarterectomy rates. At the time of acute PE diagnosis the absence of major transient risk factors, CT features of pulmonary hypertension and chronic thromboembolism are risk factors for a subsequent diagnosis of CTEPH.

## Introduction

Pulmonary embolism (PE) is a condition in which thrombus, usually embolised from the veins of the pelvis or lower limbs, obstructs the pulmonary arterial vascular bed. The incidence of PE is estimated at 60-70 per 100,000 per year [1] with a 1-year mortality of 15% [2, 3]. In survivors, patency of the pulmonary vasculature is restored, in most patients, within the first few months. [4] However, pulmonary emboli may not resolve and patients may also develop a chronic obstructing microvasculopathy.[5, 6] This can lead to elevation of pulmonary artery pressure and if untreated, the development of progressive right heart failure.[7] Once a life-limiting condition, the management of chronic thromboembolic pulmonary hypertension (CTEPH), has been transformed by the development of a multimodal treatment approach including pulmonary endarterectomy (PEA), balloon pulmonary angioplasty (BPA) and pulmonary vasodilator therapy. [8–10]

Consequently, there is interest in strategies to diagnose CTEPH. [8, 11–13] Published data notes an incidence of CTEPH following acute PE of 0.1-9.1% [14–20], reflecting differences in cohorts studied, study design and the tools used to establish the diagnosis of CTEPH. In a meta-analysis the incidence of CTEPH following PE was estimated at 0.56% in 2 studies of “all comers” and in another cohort that included only survivors the incidence was 3.21% and 2.78% in survivors without major comorbidities[21]. Recently, a large prospective, multicentre, observational cohort study, enrolled 1017 patients with acute PE and followed these patients with a standardised assessment at 3,12 and 24 months including a clinical assessment, natriuretic peptides, exercise testing and echocardiography. In that study CTEPH was diagnosed in 16 (1.6%) of patients with an estimated 2-year cumulative incidence of 2.3%.[22] If one were to extrapolate from the incidence of acute PE one would expect rates of CTEPH diagnosis of up to 15 cases per million/year.[1] However, observed rates of CTEPH diagnosis from data reported in literature from the UK and other European countries show observed rates of CTEPH diagnosis to be 4-7 cases per million/year [23] and pulmonary endarterectomy rates of 0.9-1.7 per million/year. [24] Consequently, there has been interest in developing strategies to increase diagnostic rates for CTEPH and to consider early detection approaches in at risk populations.[25, 26] ESC/ERS guidelines on PE [13] recommend that patients should be systematically evaluated following acute PE to assess for CTEPH. However, there is no consensus on how patients should be followed-up after an episode of acute PE. Algorithms for predicting CTEPH [27] or ruling out CTEPH [28] have been proposed but have not been widely incorporated into clinical practice.

The primary aim of this study was to assess the impact of an integrated acute pulmonary embolism pathway, in a large volume tertiary referral centre with expertise in acute PE and the management of CTEPH and assess whether such a pathway can achieve high CTEPH diagnostic rates in a real-world setting.

## Methods

Consecutive patients who attended a dedicated follow-up clinic after an acute episode of PE at Sheffield Teaching Hospitals NHS Foundation Trust and consecutive patients from the ASPIRE Registry (Assessing the Spectrum of Pulmonary hypertension Identified at a REferral Centre) diagnosed with CTEPH at the Sheffield Pulmonary Vascular Disease Unit between March 2010 and March 2020 were included. The ASPIRE Registry includes data on consecutive patients undergoing investigation at a pulmonary hypertension (PH) referral centre based in Sheffield, UK from 2001 onwards [29]. Data collected as part of routine clinical care is stored within hospital clinical systems is then exported and anonymised, prior to analysis. During assessment patients undergo systematic evaluation including multimodality imaging and right heart catheterisation in accordance with annually audited national standards of care [25].

CTEPH was defined according to the recommendations of 2022 ESC/ERS PH guidelines [8] and required supportive imaging, a mean pulmonary artery pressure (mPAP) >20 mmHg and PVR >2 Wood units at right heart catheterisation (RHC), with other causes of PH excluded. In occasional cases RHC data was not available, for example in patients who declined or where co-morbidities made invasive investigations inappropriate. In these cases, a diagnosis of CTEPH was made by multimodality imaging and expert opinion.

### The Sheffield PE Service and PE follow-up clinic

**Figure 1:**
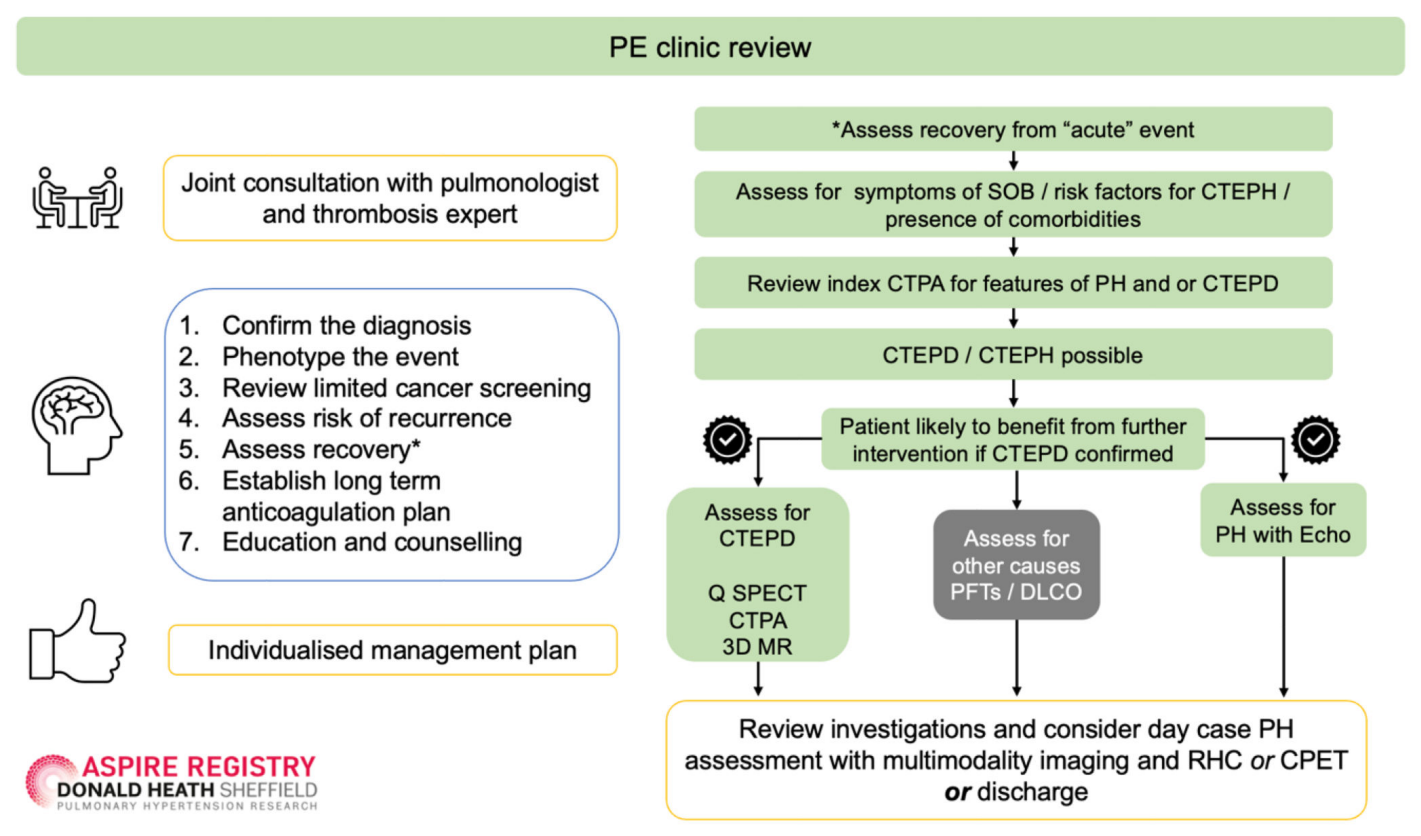
Acute PE follow-up pathway and assessment for suspected CTEPH Abbreviations. PE= pulmonary embolism; SOB= shortness of breath; CTEPH= chronic thromboembolic pulmonary hypertension; CTEPD= chronic thromboembolic pulmonary disease**;** Q SPECT= Perfusion single-photon emission computerised tomography**;** CTPA= computerised tomography pulmonary angiogram; 3D MR= 3 dimensional magnetic resonance imaging; PFT= pulmonary function tests; DLco: diffusing capacity of the lungs for carbon monoxide**;** Echo= echocardiogram**;** RHC= right heart catheterisation; CPET: cardiopulmonary exercise testing. Note patients also receive a follow-up appointment with a thrombosis nurse specialist within the first week of discharge. To assess for CTEPD patients underwent Q SPECT or CTPA or less frequently 3DMR imaging.

From 2010 onwards, physicians managing Sheffield residents discharged with a new diagnosis of acute PE, were encouraged to refer patients for follow-up in the PE service, with the exception of patients with malignancy (who were managed in a separate clinical pathway) and patients who were deemed by the referring physician to be frail, where decisions with respect to anticoagulation were managed by the thrombosis team. Patients were assessed within 1 week of discharge in an anticoagulation specialist nurse led clinic which included a clinical assessment and malignancy screening. If concerns were identified patients were discussed at a thrombosis MDT. Otherwise, patients were reviewed 3-4 months after their acute PE in a joint clinic, with a pulmonologist specialising in PE and a haematologist specialising in thrombosis. Patients were evaluated with respect to risk of venous thromboembolism (VTE) recurrence, duration of anticoagulation and assessed for possible CTEPH (Figure 1). CTEPH screening and early detection included a review of the clinical presentation and diagnostic imaging, assessment of symptoms and review of risk factors for CTEPH. In selected cases, further investigation for CTEPH including an assessment of lung perfusion and assessment of pulmonary artery pressure (Figure 1) was performed. Following this initial assessment selected patients with suspected PH were referred to the Sheffield PH referral centre where they underwent systematic evaluation with multimodality imaging (lung scintigraphy, CT pulmonary angiography, cardiac magnetic resonance (MR) with MR angiography and where there remained diagnostic doubt digital subtraction angiography) and RHC. Patients who were recovered from their acute PE or investigated and found not to have CTEPH were discharged with advice to represent for assessment if they developed symptoms of CTEPH. Unless the patient declines, all cases of CTEPH are referred to the UK national referral centre for PEA and BPA (Royal Papworth Hospital). [30] In order to establish an estimate of the number of patients diagnosed with acute PE in the Sheffield area during the duration of the study, all CTPA reports from the Sheffield 3D lab were evaluated from March 2010 to March 2020 and patients with a diagnosis of PE from a Sheffield post-code were identified.

### Features of CTEPH on CTPA performed at the time of the index PE in Sheffield PE patients diagnosed with CTEPH at follow-up

For patients diagnosed with CTEPH attending the Sheffield PE clinic, CTPAs were retrieved and reviewed by a consultant radiologist experienced in pulmonary vascular disease. The presence or absence of CT features predictive of the presence of PH and CTEPD were recorded. Where all 3 features of PH were present; PA size ≥30mm, right ventricular outflow hypertrophy ≥6mm and right ventricular:left ventricular ratio ≥1 and at least 2 of 4 features of chronic thromboembolic pulmonary disease (CTEPD) (dilated bronchial arteries, arterial webs or bands, attenuated or occluded vessels and mosaic parenchymal perfusion pattern) were present, the patient was defined as having CTEPH at the time of the initial presentation. A control group of randomly selected patients who were not diagnosed during follow-up with CTEPH were also examined for features of PH and CTEPD at the time of the index PE.

### Patient Groupings

All patients diagnosed with CTEPH during the study period were divided into 3 groups according to the origin of referral: Sheffield PE clinic, Sheffield residents referred with suspected PH, and non- Sheffield residents referred with suspected PH. Sheffield residents were defined as those who lived in a postcode area of the City of Sheffield. For the 3 patient groups, demographic, investigation and survival data were retrieved from the ASPIRE registry.[29]

### Statistical Analysis

Statistical analysis was performed using IBM SPSS Statistics v26 (SPSS, Chicago, IL, USA). Continuous variables were described by mean ± standard deviation (SD) and data that was not normally distributed are shown as median ± interquartile range (IQR). Kruskal-Wallis statistical test with Bonferroni correction was used to compare the 3 groups. Event (death)-free survival from the date of diagnosis was estimated using the Kaplan-Meier method with comparison between groups performed by the log-rank test. A p-value <0.05 was deemed statistically significant.

### Population data

Rates for annual incidence were based on a mean population of the city of Sheffield, derived from the 2011 and 2021 department of national statistics census data, of 554,600. The Sheffield PH referral centre is part of a UK national network, adhering to annually audited and published standards of care, and covers a referral population of 15–20 million. Ethical approval for this study was obtained through the ASPIRE registry (REC 22/EE/0011).

## Results

### Population

Between March 2010 and March 2020, 1956 patients attended the PE clinic and 850 patients from the ASPIRE Registry were diagnosed with CTEPH, 41 from the Sheffield PE clinic and 809 referred with suspected pulmonary hypertension. PE clinic patients were followed up within this study for a median of 66 (IQR 55) months. Demographic and baseline clinical characteristics of patients diagnosed with CTEPH, including those undergoing PEA, are shown in Table 1. Sheffield patients with CTEPH diagnosed at the PE clinic were older with a better exercise capacity than patients referred with PH (Tables 1 and 2).

### Patients diagnosed with acute PE in Sheffield following CTPA

During the conduct of this study 20,494 patients from Sheffield underwent CTPA, 3289 patients (16%) were diagnosed with PE and1956 (59%) of these patients were seen in the PE clinic.

### Sheffield acute PE follow-up clinic

The 1956 patients reviewed in the PE clinic were seen at a mean of 3.95±1.94 months following a diagnosis of acute PE and where CTEPH suspected were investigated as per Figure 1. Between 2010-2020, 120 patients seen in the PE clinic were evaluated at the Sheffield PH referral centre. Of these, 41 were diagnosed with CTEPH, 7 PH associated with respiratory disease, 6 PH associated with left heart disease, 2 pulmonary arterial hypertension (1 congenital heart disease, 1 associated with connective tissue disease), 62 had no PH (39 of these patients were classified as CTEPD non-invasively and 15 of these patients were diagnosed with CTEPD with a RHC) and in 2 patients no final diagnosis was possible.

**Figure 2:**
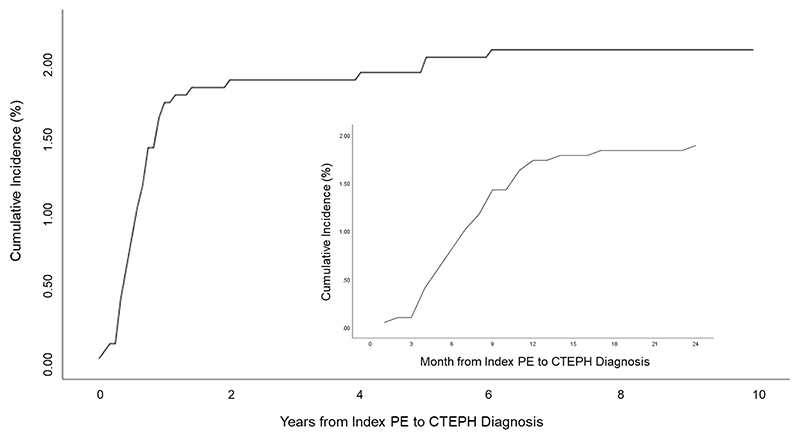
Total and 2-year cumulative incidence of chronic thromboembolic pulmonary hypertension from index pulmonary embolism event (n=1956) Abbreviations. PE=pulmonary embolism; CTEPH=chronic thromboembolic pulmonary hypertension

The cumulative incidence of developing CTEPH from an index PE over the 10-year study period was 2.1%. CTEPH diagnosis was confirmed in 51% within 4 months, 71% within 6 months, 88% within 12 months and 90% within 24 months of first PE clinic appointment. The cumulative incidence of CTEPH diagnosis from the incident PE event is shown in Figure 2. The estimated 2-year cumulative incidence from the index PE event was 1.89%. Over the 10-year period a single patient was discharged with a non-invasive diagnosis of chronic thromboembolic pulmonary disease (CTEPD) but subsequently represented and was diagnosed with modest haemodynamic CTEPH 4.3 years later.

**Figure 3:**
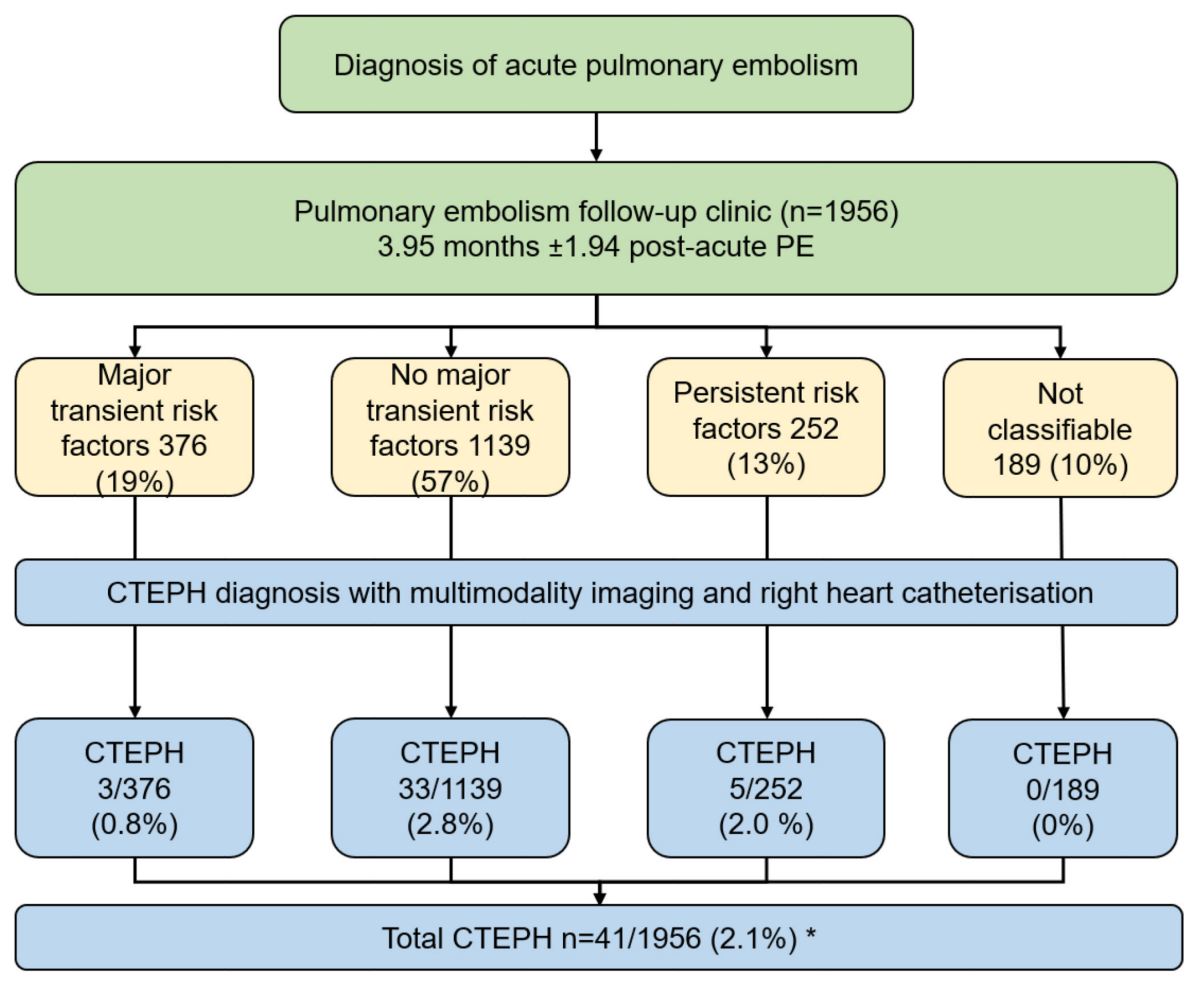
Flow chart showing acute PE pathway and impact of risk factors for acute PE on diagnosis of CTEPH Abbreviations. PE= pulmonary embolism; CTEPH=chronic thromboembolic pulmonary hypertension. *All patients diagnosed at RHC apart from 2 patients who declined RHC, diagnosis in these cases was made based on multimodality imaging and expert opinion.

Patients with no major transient risk factors present at the time of acute PE had a significantly higher risk of CTEPH compared to patients with major transient risk factors, OR of 3.6 (95% CI 1.11 to 11.91, p=0.03), however, there was no significant difference between patients with persistent risk factors and those with major transient risk factors OR of 2.49 (95% CI 0.59-11.50, p=0.22), Figure 3.

At the time of CTEPH diagnosis all patients had received at least 3 months of anticoagulation with 26 patients (63%) receiving warfarin, 13 (32%) a direct oral anticoagulant (DOAC) and 2 (5%) low molecular weight heparin. From 2015 onwards, 13 (54%) received a DOAC, 10 (42%) received warfarin and 1 (4%), low molecular weight heparin.

**Figure 4:**
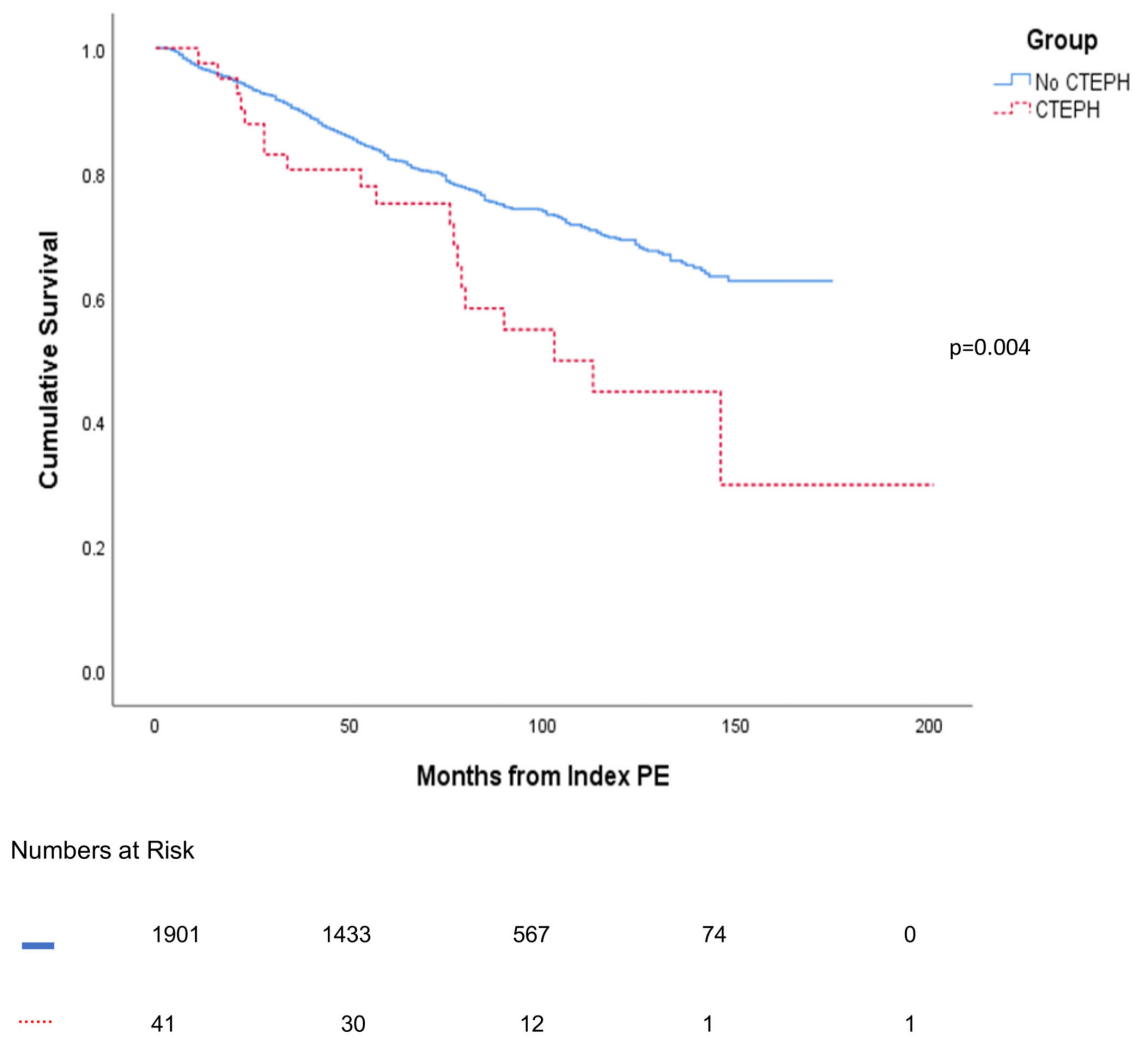
Kaplan-Meier Survival curve for patients who attended the pulmonary embolism clinic split into those diagnosed or not diagnosed with CTEPH Abbreviations. CTEPH= chronic thromboembolic pulmonary hypertension; PE= pulmonary embolism

Patients from the PE clinic diagnosed with CTEPH had a significantly worse survival compared to patients not diagnosed with CTEPH; the 1, 3 and 5-year survival for patients attending the PE clinic with CTEPH versus those without CTEPH were 95% vs 96%, 78% vs 89% and 73% vs 83%, respectively, p=0.004, Figure 4. There was no significant difference in survival for patients undergoing PEA diagnosed in the PE clinic compared to those presenting with suspected PH with 1 and 3-year survival of 100% vs 95% and 100% vs 89%, respectively p=0.08.

### Signs of pre-existing CTEPD and CTEPH on the CTPA at the time of the index PE in patients diagnosed with CTEPH from the Sheffield PE clinic

**Figure 5:**
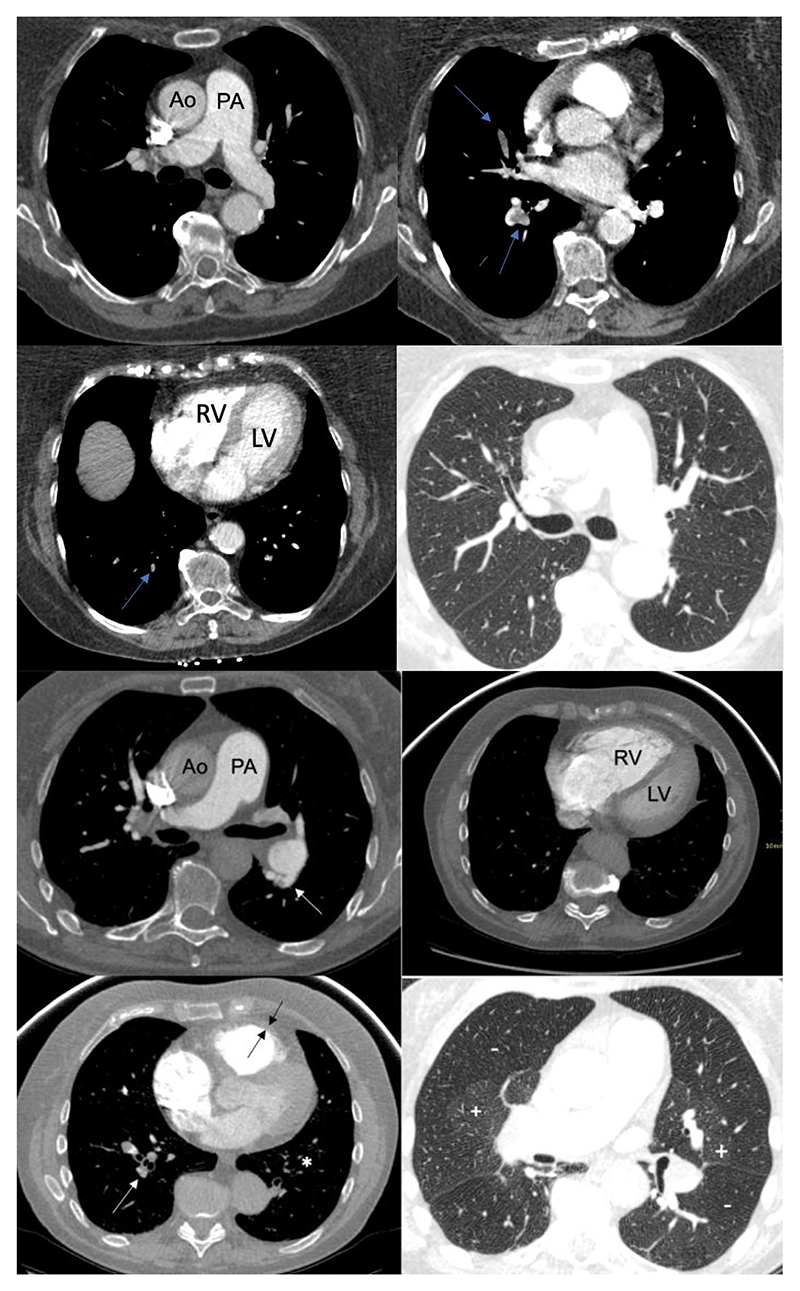
CTPAs from the Sheffield PE Clinic from the index PE in a patient who did not go on to develop CTEPH (Top) and a patient who went on to develop CTEPH (Bottom) Figure 5 Top: of a patient from the time of the index PE who did not go on to develop CTEPH; note mild PA dilation, but a RV:LV ratio <1, no RVOTH and no features of CTEPD, filling defects shown by blue arrows. Figure 5 Bottom: of a patient from the time of the index PE who went on to develop CTEPH; note all 3 features of pulmonary hypertension: PA≥30mm, RVOTH≥6mm (between black arrows) and RV:LV≥1 and 3 features of CTEPD; arterial webs (white arrows) attenuated vessels (white asterix) and mosaic parenchymal perfusion pattern + and -). Ao: aorta; CTEPD: chronic thromboembolic pulmonary disease; CTEPH: chronic thromboembolic pulmonary hypertension; LV: left ventricle; PA:pulmonary artery; RV:right ventricle; RVOTH: right ventricular outflow tract hypertrophy

CTPAs from the index PE were available for 36 of 41 patients diagnosed with CTEPH from the Sheffield PE clinic and features of CTEPD and PH were compared to a control group of 36 patients from the Sheffield PE clinic not diagnosed with CTEPH during the follow-up period (Table 3). Two or more features of CTEPD and all 3 features of PH were present in 12 (33 %) and 13 (36%) of patients diagnosed with CTEPH compared to 1 (3%) and 2 (6%) of patients not diagnosed with CTEPH. Features defined as indicative of CTEPH at diagnosis (≥2 CT features of CTEPD and all 3 CT features of PH) were present in 7 (19%) of patients diagnosed with CTEPH compared to 0 (0%) of patients not diagnosed with CTEPH during follow-up. Figure 5, shows illustrative imaging from the time of the index PE event from a patient who went on to develop CTEPH during the study period compared to a patient who did not.

### Characteristics of patients diagnosed with CTEPH including those undergoing pulmonary endarterectomy

**Figure 6:**
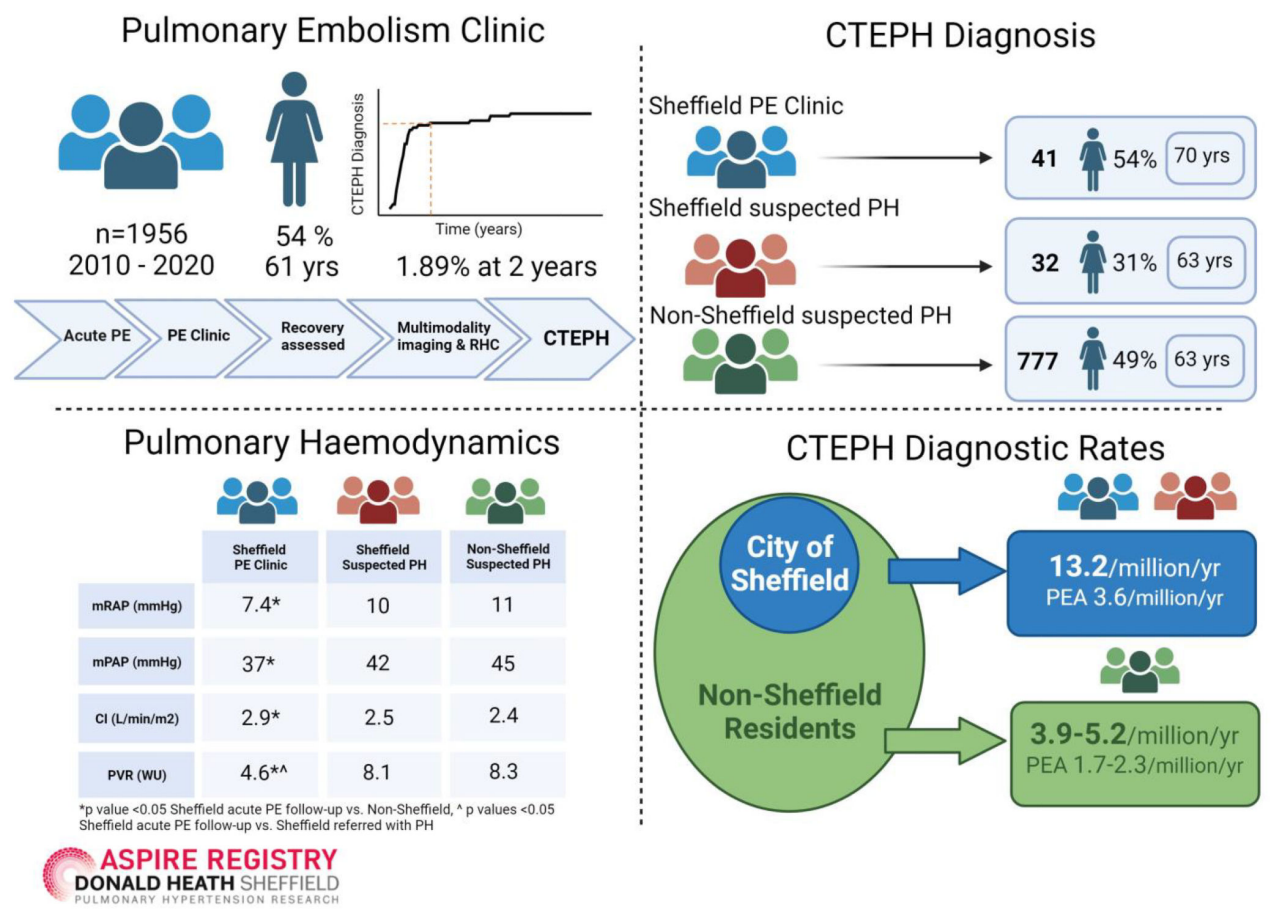
Central figure illustrating key results including population-based CTEPH diagnostic rates and pulmonary endarterectomy rates Abbreviations. PE= pulmonary embolism; RHC= right heart catheterisation; CTEPH= chronic thromboembolic pulmonary hypertension; PH= pulmonary hypertension; mRAP= mean right atrial pressure, mPAP= mean pulmonary artery pressure; CI= cardiac index; PVR= pulmonary vascular resistance. Created with BioRender.

A total of 850 patients from the ASPIRE Registry were diagnosed with CTEPH over the 10-year study period. Seventy-three of these patients originated from the city of Sheffield. The diagnostic rate for residents from Sheffield with CTEPH was 13.2/million/year (based on a referral population of 544,600). Diagnostic rates were significantly lower for non-Sheffield residents at 3.9–5.2/million/year (based on a referral population of 15-20 million), (Figure 6).

Of 73 Sheffield patients, 41 were referred from the PE clinic and 32 were referred directly with suspected PH. Of Sheffield patients directly referred with suspected PH, 26 (81%) were referred from specialist out-patient clinics, primarily cardiology and respiratory and 6 (19%) were referred as in-patients, usually with decompensated right heart failure. Of the 32 patients referred with suspected PH from Sheffield, 11 had admissions with acute PE within the study period but did not attend the PE clinic. CT scans were available for 10 of these patients, of whom 5 (50%) had features of PH, 6 (60%) had 2 or more features of CTEPD and 3 (30%) had both 3 features of PH and 2 or more features of CTEPD.

Patients diagnosed with CTEPH from the PE clinic had a shorter duration of symptoms preceding their diagnosis of CTEPH (median 6 months (IQR 7)) than those referred with suspected PH (median 12 months (IQR 12) p<0.01). Patients diagnosed with CTEPH from the PE clinic also had less severe pulmonary haemodynamic disease (pulmonary vascular resistance 4.6±2.4 WU versus 8.3±4.6 WU, p<0.01) and significantly better exercise tolerance (incremental shuttle walking distance 305±218m versus 200±189m, p<0.05) than those referred with PH from outside Sheffield (Table 1). There was no significant difference in pulmonary haemodynamics between Sheffield and non-Sheffield residents referred with suspected PH (p>0.05, Table 1).

Forty-four percent of non-Sheffield residents referred with suspected PH who were diagnosed with CTEPH underwent PEA surgery, whereas 27% of Sheffield residents diagnosed with CTEPH were operated. Nonetheless, population-based PEA rates were significantly higher for Sheffield residents at 3.6/million/year compared to 1-7-2.3 /million/year for non-Sheffield residents. Balloon pulmonary angioplasty was commissioned as a national service in the UK in 2018 and at the census date no patients from the Sheffield PE clinic had undergone BPA.

## Discussion

In the real world setting we have shown that the introduction of a dedicated PE follow-up pathway identifies patients with a shorter duration of symptoms, less exercise limitation and less severe CTEPH than patients presenting with PH, whilst increasing population-based CTEPH diagnostic and PEA rates. Specifically, we have shown that diagnostic rates for CTEPH of 13.2/million/year and PEA rates of 3.6/million/year are achievable. In addition, at the time of acute PE diagnosis the absence of major transient risk factors for thromboembolism and CT features of PH and chronic thromboembolism are risk factors for a subsequent diagnosis of CTEPH.

### Incidence and outcome of CTEPH following acute PE

In this study patients received a gold standard diagnosis of CTEPH, undergoing multimodality imaging, RHC and multidisciplinary team discussion in a centre experienced in CTEPH management. Our population was unselected, consecutively enrolled and therefore representative of real-world data with a significantly longer duration of follow-up than most previous studies. The cumulative incidence of CTEPH reported in the literature ranges from 0.1- 9.1%.[14–17, 21] Recently, the FOCUS study reported a 2-year incidence of CTEPH of 2.3% which is similar to the 1.89% reported in this study.[22] We have also observed, for the first time, that patients diagnosed with CTEPH from the index PE event, have a significantly worse survival than patients without CTEPH, highlighting the importance of early assessment for CTEPH following acute PE (Figure 4).

### Population-based CTEPH diagnostic and PEA rates

By employing an integrated pathway for acute PE follow-up we observed high population-based diagnostic rates for CTEPH (13.2/million/year) in Sheffield residents, higher than the 3.9-5.2/million/year in non-Sheffield residents and 4-7/million/year reported in an epidemiological analysis from Europe, Japan and USA[24, 31]. The population of patients diagnosed with CTEPH from the PE clinic were older with more comorbidities than patients referred with suspected PH, however they also had a better exercise capacity and less severe pulmonary haemodynamics. Our results suggest if a similar standardised approach to acute PE follow-up was adopted elsewhere then one would expect to see CTEPH diagnostic rates 2-3-fold higher than currently observed. For Sheffield residents we also report high population-based rates for PEA at 3.6/million/year, significantly higher than the 0.9/million/year PEA rate estimated in the USA, 1.7/million/year in Europe [24, 31] and 2.2/million/year noted in the UK national audit of PH during the duration of this study 2010-2020[25].

### Characteristics and management of CTEPH identified in the PE clinic versus patients referred with suspected PH

Although we observed PEA population-based rates for Sheffield residents during the 10-year duration of this study to be significantly higher than previously published international rates, a significantly lower proportion of Sheffield residents with CTEPH from the PE clinic (27%) proceeded to PEA than non-Sheffield residents referred with suspected PH (44%). Patients attending the PE clinic had milder haemodynamic abnormalities and despite being older, a better exercise capacity than patients referred with suspected PH. It is likely these factors impacted on the 39% of patients who declined surgery. Where strategies are in place to identify patients with CTEPH following PE, our data suggests that significant numbers of patients, particularly those who are older with comorbidities, are more likely not to undergo PEA. We have previously shown that patients with surgical disease who decline surgery have a worse prognosis than patients who undergo pulmonary endarterectomy, [32] highlighting that further work needs to be performed to understand factors influencing treatment decisions and identify the most appropriate regimen tailored to the individual patient. Our data also demonstrates the importance of how we model data to estimate the need for surgical provision. Although we can achieve CTEPH diagnostic rates 2-3-fold higher than currently reported, in the UK where population-based endarterectomy rates are the highest in the world, it is likely that increases in surgical capacity will be more modest.

### Sheffield PE follow-up clinic

Our data supports a targeted approach whereby patients are carefully assessed at 3-4 months following acute PE, (Figure 1). Those who are at low risk of CTEPH or symptomatically well are discharged with advice and asked to represent if increasingly symptomatic. We also confirm the findings of a previous study [21] that patients with no major transient risk factors present at the time of the index acute PE have a significantly higher risk of CTEPH. Computed tomography features of CTEPD and CTEPH were frequently seen at diagnosis in patients who developed CTEPH during follow-up, confirming the results of a smaller previous study which identified features of CTEPD in 4 out of 7 patients subsequently diagnosed with CTEPH [20]. This highlights the importance of reviewing the CTPA at the time of the index PE in addition to identifying risk factors for the development of CTEPH. Whereas previous investigators have focussed on features of CTEPD at the index PE (dilated bronchial arteries, arterial webs or bands, attenuated blood vessels and mosaic parenchymal perfusion pattern) we have shown the value of also looking at CT features of pulmonary hypertension. All 3 CT features of PH were present (PA≥30mm, RV:LV ratio≥1 and RVOTH≥6mm) in combination with at least 2 out of 4 features of CTEPD in 19% of patients who developed CTEPH and in 0% of patients who did not develop CTEPH at follow-up. Whereas enlargement of the PA and an increase in RV:LV ratio were seen in 67% and 78% of patients who developed CTEPH compared to 31% and 36% of patients who did not develop CTEPH, respectively, RVOTH and all 3 features of PH were present in 44% and 36% of patients who developed CTEPH and in only 8% and 6% of patients, suggesting that the presence of RVOTH and all 3 features of PH may aid identification of patients at significantly increased risk of developing CTEPH. These CT features are easier to appreciate by the non-specialist radiologist and are also amenable to automated analysis using AI approaches. [33] In contrast, features of CTEPD are more challenging to identify by the non-specialist and to date have eluded automated diagnosis using AI approaches. A standardised approach to acute PE follow-up also allows for the introduction of long-term anticoagulation for those at increased risk of recurrent VTE in addition to providing counselling and education for patients (Figure 1). Long-term anticoagulation would be expected to reduce recurrent VTE and may reduce the subsequent risk of CTEPH, if offered to suitable patients with no major transient risk factors for their acute PE.

### Limitations

This study is limited by its single centre and retrospective design and the findings would benefit from external validation. A significant number of patients (41%) diagnosed with acute PE during the conduct of this study were not followed up in the PE clinic; this included patients with malignancy who were followed in a separate pathway and those deemed to be frail by the referring physician. It is possible that some of these patients may develop CTEPH in the future and present with PH. In addition, some patients seen in the PE clinic may have become symptomatic and not sought medical advice and have undiagnosed CTEPH. Nonetheless, our results do establish minimum population-based rates of CTEPH diagnosis and PEA that are likely representative of those that can be achieved in large metropolitan districts. Our PE clinic includes both a thrombosis expert and a respiratory physician and the results may not be directly translatable to alternative types of PE clinic provision.

## Conclusion

In conclusion, in the largest study to report on CTEPH diagnostic rates in patients undergoing systematic assessment following an acute PE, we have demonstrated that a single follow-up visit can identify patients earlier with less severe CTEPH and can achieve higher population based CTEPH diagnostic and pulmonary endarterectomy rates that seen in previous studies. In addition, at the time of the index PE event the absence of major transient risk factors for venous thromboembolism and CT features of PH and chronic thromboembolism, should alert the physician to the possibility of CTEPH. This study adds to the growing evidence supporting structured follow-up of patients with venous thrombo-embolic disease.

## Figures and Tables

**Table 1:** Baseline characteristics of patients diagnosed with chronic thromboembolic pulmonary hypertension (CTEPH) and undergoing pulmonary endarterectomy (PEA)

Demographic Data	Sheffield *		Non-Sheffield ^§^
	PE clinic	Referred with suspected PH^+^	Referred with suspected PH
**All patients**			
**CTEPH (n)**	41	32	777
**Age at diagnosis (Years)**	69.46 ± 14.49^¥^	63.38 ± 12.73	63.44 ± 4.95
**Sex: Female (%)**	22 (54)	10 (31)	382 (49)
**WHO FC I/II/III/IV (%)**	0/27/71/2	3/9/75/13	1/8/82/9
**ISWT (m)**	305 ± 218^¥^	221 ± 210	200 ± 188.30
**mRAP (mmHg)**	7.4 ± 3.9^¥^	10 ± 5.1	11 ± 6.0
**mPAP (mmHg)**	37 ± 10.1^¥^	42 ± 13.9	45 ± 11.5
**PAWP (mmHg)**	12 ± 3.7	12 ± 3.9	11 ± 6.09
**CI (L/min/m2)**	2.87 ± 0.65^¥^	2.54 ± 0.81	2.40 ± 0.76
**PVR (Wood Units)**	4.6 ± 2.4^#¥^	8.1 ± 6.5	8.3 ± 4.6
**SvO_2_ (%)**	59.4 ± 24 ^#¥^	62.9 ± 7.6	56.3 ± 19.09
**Patients undergoing PEA**			
**PEA (n)**	11	9	344
**Age at diagnosis (years)**	67.36 ± 12.01^#¥^	56.5 ± 13.44	58.11 ± 14.6
**Sex: Female (%)**	8 (73)	4 (50)	153 (44)
**WHO FC I/II/III/IV (%)**	0/45/55/0	0/25/74/0	0/8/86/5
**ISWT (m)**	360 ± 182	267 ± 163.4	241 ± 187
**mRAP (mmHg)**	7 ± 2.7	12 ± 6.05	10 ± 6.6
**mPAP (mmHg)**	41 ± 10.4	47 ± 9.8	46 ± 11.5
**PAWP (mmHg)**	11 ± 3.6	10.4 ± 6.6	11.9 ± 5.23
**CI (L/min/m2)**	3.1 ± 0.7^¥^	2.44 ± 0.75	2.42 ± 0.66
**PVR (Wood Units)**	5.4± 2.4	10.0 ± 5.8	8.29 ± 4.6
**SvO_2_ (%)**	70 ± 4.8^#¥^	58 ± 7.51	62.3 ± 8.79

Abbreviations. PE= pulmonary embolism; PH= pulmonary hypertension; CTEPH= chronic thromboembolic pulmonary hypertension; WHO FC= world health organisation functional class; ISWT= incremental shuttle walk test; mRAP= mean right atrial pressure, mPAP= mean pulmonary artery pressure; PAWP= pulmonary arterial wedge pressure; CI= cardiac index; PVR= pulmonary vascular resistance; PEA= pulmonary endarterectomy.

* Sheffield patients were defined as a Sheffield resident according to their residing postcode being within the city of Sheffield as per Sheffield City council.

§ Non-Sheffield patients were those residing outside the postcodes classed as the City of Sheffield.

¥ p values <0.05 Sheffield acute PE follow-up vs. Non-Sheffield,

# p values <0.05 Sheffield acute PE follow-up vs. Sheffield referred with PH.

+ out-patient referrals (n=26, 81%), in-patient hospital transfers (n=6, 19%).

**Table 2:** Baseline characteristics of Sheffield residents diagnosed with CTEPH from the pulmonary embolism clinic compared to those referred with suspected pulmonary hypertension

		Sheffield	
		PE clinic	Referred with suspected PH
**CTEPH (n)**		41	32
**Age at diagnosis (Years)**		69.46 ± 14.49	63.38 ± 12.73
**Sex: Female (%)**		22 (54)	10 (31)
**WHO FC I/II/III/IV (%)**		0/27/71/2	3/9/75/13
**Risk factors for venous thromboembolism (%)**	**History of venous thromboembolism**	41 (100)*	12 (39)^+^
	**Thyroid disease**	5 (12)	1 (3)
	**Cancer or myeloproliferative disease**	4 (10)	7 (22)
	**Hormonal therapies**	1 (2)	1 (3)
	**Splenectomy**	1 (2)	1 (3)
	**Pregnancy or puerperium**	0	0
**Co-morbidities (%)**	**Chronic heart failure or coronary heart disease or valvular disease**	12 (29)	5 (16)
	**Systemic hypertension**	11 (27)	2 (6)
	**Diabetes mellitus**	8 (20)	3 (9)
	**Atrial Fibrillation**	5 (12)	2 (6)
	**Chronic obstructive pulmonary disease**	5 (12)	6 (19)
	**Chronic renal failure**	5 (12)	3 (9)
	**Chronic liver disease**	0	1 (3)
**Symptom duration (%)**	**<1 year**	76	38
	**1-2 years**	12	28
	**>2 years**	7	19
	**Not clear**	5	16
**CTEPH operated (%)**		11 (27)	9 (28)
**CTEPH not operated (%)**	**Disease distribution**	2 (5)	2 (6)
	**Co-morbidities**	6 (15)	8 (25)
	**Mild disease**	4 (10)	5 (16)
	**Patient choice**	16 (39)	5 (16)
	**Not clear**	1 (2)	1 (3)
	**Died**	1 (2)	2 (6)

PE=pulmonary embolism; PH= pulmonary hypertension; CTEPH= chronic thromboembolic pulmonary hypertension; WHO FC= world health organisation functional class.

* 14 (34%) of the patients diagnosed with CTEPH from the acute PE follow-up clinic had a previous history of VTE prior to their diagnosis with acute PE

+ 11 patients (2 after 2015) presented with a PE between 2010 and 2020 and were not referred to the PE Clinic but were subsequently referred directly to the PH referral centre from hospital out-patient clinics (n=9), as a hospital in-patient transfer (n=1) and from primary care (n=1).

**Table 3:** Features present on initial CTPA at time of PE diagnosis in patients with subsequent diagnosis of CTEPH and a randomly selected control group of patients not diagnosed with CTEPH

	CTEPH +ve (n=36)	CTEPH -ve (n=36)
**Demographics:**		
Age, years (mean ± SD)	69.9 ± 13.9	68.5 ± 11.95
Sex M/F n, (%)	18 (50)/18 (50)	16 (44)/20(56)
**Features suggestive of PH:**		
PA ≥ 30mm n, (%)	24 (67)	11 (31)
RV:LV ≥1 n, (%)	28 (78)	13 (36)
RVOTH ≥6mm n, (%)	16 (44)	3 (8)
All 3 features suggestive of PH present n, (%)	13 (36)	2 (6)
**Features suggestive of CTEPD:**		
Dilated bronchial arteries, yes/no (%)*	10 (28)	2 (6)
Arterial webs or bands, yes/no (%)	4 (11)	1 (3)
Attenuated or Occluded vessels combined, n (%)	13 (36)	1 (3)
Mosaic parenchymal perfusion pattern yes/no n, (%)	8 (22)	2 (6)
2 or more features suggestive of CTEPD n, (%)	12 (33)	1 (3)
**Features suggestive of CTEPH at index event:**		
3 features of PH and ≥ 2 features of CTEPD n, (%)	7 (19)	0 (0)

PA=pulmonary artery; RV:LV=right to left ventricular ratio; RVOTH=right ventricular outflow tract hypertrophy; PH=pulmonary hypertension; CTEPD=chronic thromboembolic pulmonary disease; CTEPH=chronic thromboembolic pulmonary hypertension.

CTPA at the time of diagnosis of sufficient diagnostic quality were available for review in 36/41 of CTEPH +ve patients and are compared to a randomly selected control group of 36 patients not diagnosed as having CTEPH during the conduct of the study. PA pulmonary artery diameter; RV:LV ratio of internal right ventricular to left ventricular diameter; RVOTH thickness of right ventricular outflow tract.

* assessment of bronchial artery size was not possible due to lack of contrast opacification in 9 (25%) of CTEPH +ve patients and 6 (17%) of CTEPH -ve patients

## Take home message

Systematic follow-up after acute pulmonary embolism at 3 months, increases population-based rates of both chronic thromboembolic pulmonary hypertension (CTEPH) diagnosis and pulmonary endarterectomy, identifying patients earlier with less severe disease. The absence of major transient risk factors for pulmonary embolism and CT features at diagnosis predict the development of CTEPH.
